# IL-6: The Link Between Inflammation, Immunity and Breast Cancer

**DOI:** 10.3389/fonc.2022.903800

**Published:** 2022-07-18

**Authors:** Juan Chen, Yanghui Wei, Weiqin Yang, Qingnan Huang, Yong Chen, Kai Zeng, Jiawei Chen

**Affiliations:** ^1^ Department of Medicine and Rehabilitation, Tung Wah Eastern Hospital, Hong Kong, Hong Kong SAR, China; ^2^ Department of Surgery, The Eighth Affiliated Hospital, Sun Yat-Sen University, Shenzhen, China; ^3^ School of Biomedical Sciences, The Chinese, University of Hong Kong, Hong Kong, Hong Kong SAR, China

**Keywords:** breast cancer, interleukin-6, inflammation, immune, target therapy

## Abstract

Breast cancer is one of the leading causes of mortality in females. Over the past decades, intensive efforts have been made to uncover the pathogenesis of breast cancer. Interleukin-6 (IL-6) is a pleiotropic factor which has a vital role in host defense immunity and acute stress. Moreover, a wide range of studies have identified the physiological and pathological roles of IL-6 in inflammation, immune and cancer. Recently, several IL-6 signaling pathway-targeted monoclonal antibodies have been developed for cancer and immune therapy. Combination of IL-6 inhibitory antibody with other pathways blockage drugs have demonstrated promising outcome in both preclinical and clinical trials. This review focuses on emerging studies on the strong linkages of IL-6/IL-6R mediated regulation of inflammation and immunity in cancer, especially in breast cancer.

## Introduction

Breast cancer is one of the leading diagnosed cancers in women with high mortality. According to International Agency for Research on Cancer (IARC), there were 2,261,419 women diagnosed with breast cancer in 2020 worldwide. It is a common cause of cancer-related death especially in less developed countries. Despite the recent advanced technique in breast cancer screening and early diagnosis, the high morbidity and mortality rates urge the need of investigation into the molecular mechanism of breast cancer.

Genome wide analyses have recently demonstrated thousands of mutations accumulated in breast cancer cells (1). In addition, as a multifactorial disease, the etiologies of breast cancer include not only distinct inherent factors such as genetic status, but also environmental factors such as obesity, lifestyle, and chronic inflammation (2).

Accumulating studies have been performed on the relationship between inflammation and cancer (3). It is well-accepted that inflammatory diseases could increase the risk of cancer development during tumor initiation, promotion, progression, and metastasis (3–6).

As one of the best-characterized pro-tumorigenic cytokines, IL-6 has been studied extensively for its central role in both physiological and pathological processes (7). Previous studies indicated that IL-6 regulate the pro-inflammatory and enhance monocyte infiltration at the inflammatory site during chronic inflammation (8). IL-6 responsive tissues would become resistant gradually during chronic inflammation, which correlated with high basal level of IL-6 (9, 10). IL-6 was also elevated in many solid tumors including breast cancer (11–13), which correlated with poor prognosis and metastasis (14, 15). The current review will further discuss the intricate relationship between IL-6, inflammation, and breast cancer.

## The IL-6 Signaling Pathways and Functions

### The Il-6 Signaling Pathway

Human IL-6 is a 26 kDa glycoprotein known as a B-cell differentiation regulator (16) which is secreted by a number of cells (17). IL-6 is a multifunctional cytokine that plays both pro-inflammatory and anti-inflammatory roles in humans (18). IL-6 is a single chain phosphorylated glycoprotein consisting of four helix bundles (A-D), with A and B run in one direction while C and D run in the opposite direction. IL-6 transmits its signals through a cell-surface type-I receptor complex, which consists of the membrane-bound IL-6 receptor (IL-6R) and a signal-transducing component gp130 homodimer (19). IL-6R is expressed on a limited number of cell types, such as macrophages, B cells and subtypes of T cells (20, 21). IL-6R is 80 kDa α-chain and is also called as CD126 consisting of three domains namely D1, D2 and D3. Besides the membrane bound receptor (mIL-6R) as previously mentioned, soluble (sIL-6R) is the other form of IL-6R, which is expressed mainly in hepatocytes, neutrophils, monocytes, and T-cells (22). IL-6 selectively activates different signaling pathways, the classical signaling pathway through mIL-6R, and the trans-signaling pathway through sIL-6R. In both the cases, IL-6 binds to the receptor and then to gp130, but elicits different biological effects depending upon the receptor form (23). Cytokine IL-6 triggers the anti-inflammatory responses through classic signaling by binging to mIL-6R and gp130, while in contrast, trans-signaling can be manifested in all gp130-expressing cells, and leads to pro-inflammatory responses (24). The sIL-6R can be found at circulation with concentration from 25 to 35 ng/ml in human, which is generated by proteolytic cleavage of the membrane bound form IL-6R and by proteolytic cleavage of metalloproteinases gene family members, or by alternative splicing of IL-6R mRNA (25). There are three routes of the IL-6 signaling pathway. In route 1, Janus kinase (JAK) is phosphorylated and activated, subsequently activates dimerization of signal transducer and transcription-3 (STAT3) (26). In route 2, JAK activates Ras/Raf pathway, causing hyperphosphorylation of mitogen activated protein kinases (MAPK) and incudes its serine/threonine kinase activity (23). The third route involves the activation of phosphoinositol-3 kinase (PI3K)-protein kinase B (PKB)/Akt pathway (27).

### IL-6 and Immunity

IL-6 is secreted by largely plasmacytoid dendritic cells (pDCs), which is critical for differentiation from B cells to plasma cells (28). This cytokine is also a vital modulator to maintain dynamic balance between Th1 and Th2 immune cells (29). For example, IL-6 is necessary during the differentiation from Th1 to Th2 cells (30). The process was proved to interfere with IFN-γ production *via* up-regulation of suppressor of cytokine signaling 1 (SOCS1) and SOCS3 in CD4+T cells (31). Meanwhile, together with transforming growth factor-β (TGF-β), IL-6 could promote the differentiation of Th17 cells *via* activating both retinoic acid-related orphan receptor γt (RORγt) and RORα (32). It was reported that STAT3 mediated the effectiveness of IL-6 on Th17 differentiation and this cytokine could inhibit the activity of Treg cells (33). Therefore, IL-6 is regarded as the main regulator of Treg/Th17 equilibrium (34).

IL-6 also plays a vital role in early differentiation of T follicular helper cells (Tfh), the main T helper cell subtype provides support for germinal center formation, affinity maturation, and immune cells’ generation. Early BCl6+/CXCR5+/Tfh differentiation would be mostly interfered in the case of IL-6 absence which was proved to mediate by STAT1 and STAT3 (35).

Novel agents against the IL-6/IL-6R signaling pathway have been proved to be effective for some inflammatory diseases. Preclinical studies have demonstrated that IL-6 has crucial functions in inflammatory cells recruitment (36). Tumor-associated macrophages (TAMs) secreted IL-6 and plays critical role in carcinogenesis and differentiation of myeloid-derived suppressor cells (MDSCs), which gives rise to intra-tumoral inflammatory processes (37, 38). A previous study demonstrated that inhibition of NF-κB decreased the stem cell compartment, which in turn reduced blood vessel formation in breast cancer (39). In addition, high expression of IL-6R on liver cells led to recruitment of acute phase proteins (40). High expression levels of acute phase proteins including CRP, fibrinogen and serum amyloid protein A were identified during both acute and chronic disease (41, 42). Interestingly, clinical observation found that CRP levels in patients with severe bacterial infections were not elevated when IL-6 was absent (43). Further studies demonstrated that blocking IL-6 signaling by neutralizing antibody may reverse low serum level of CRP (44). However, the application of IL-6/IL-6R blockers as anti-cancer agents has not been proved intensively in cancers including breast cancer.

### IL-6 and Stem Cell

IL-6 family cytokines play an important role in generation and maintenance of stem/progenitor cells including cancer stem cells (CSCs) (45). As a member in IL-6 family, leukemia inhibitory factor (LIF) has an crucial role in both embryonic stem (ES) cells and cancer development (46), which is necessary to maintain mouse ES cells in an undifferentiated condition *via* STAT3 activation (47). Active LIF was detected in a wide range of malignancies including lung, breast, stomach, colon, liver, gallbladder, and pancreatic carcinoma (48). Once activated, STAT3 may induce gene expression including c-Myc, which contribute to the maintenance of undifferentiated state in mouse ES cells (49). It is also reported that IL-6 increased pluripotent stem (iPS) cell population by inducing c-Myc and Pim1 (50). The transcription factor C/EBPδ, was reported to be pro-tumorigenic in breast cancer cell lines by directly targeting IL-6R, leading to cancer progression with cancer stem cells activation (51). The IL-6-JAK1-STAT3 pathway has a vital function in the transition from non-CSCs into CSCs by regulating OCT4 in human breast cancer cell lines (52). In lung cancer CSCs, IL-6Rα was detected in CSCs (53), whereas STAT3 was necessary for proliferation and survival in colon cancer-initiating cells (54, 55). It was reported that constitutive activation of STAT3 and NF-κB signaling in glioblastoma CSCs regulate Notch pathway, which played a key role in CSC maintenance and cell survival (56). STAT3 activation by IL-6 from adipose-derived stem cells could promote endometrial carcinoma proliferation and metastasis (57).

IL-6 is also crucial for epigenetic modification in stem cells (58, 59). NF-κB and STAT3 were identified as key regulators in epigenetic switch in inflammation (60, 61). Recently, a positive feedback loop involving microRNA let-7 has been demonstrated for maintaining chronic inflammatory status in malignant cells (60). Interestingly, this feedback loop regulated by IL-6 signaling could in turn activate NF-κB pathway and its downstream targets such as let-7 and Lin-28. Similarly, IL-6 was proved to be essential in keeping inflammatory loop in breast cancer CSCs (60, 61). In summary, IL-6 signaling plays a regulatory role in controlling cancer cell growth, CSC renewal and metastasis (62).

### IL-6 and Tumor Microenvironment

Tumor microenvironment contributes significantly towards potentiating the stemness and metastasis properties of cancer cells. Solid tumors, including breast cancer cells were reported to have intense interaction with stromal cells such as mesenchymal stem cells (MSCs), adipocytes, cancer associated fibroblasts (CAFs), endothelial cells and immune cells in tumor microenvironment (63). Majority of these stromal cells within tumor microenvironment could secrete both IL-6 and IL-8 (63, 64). Mesenchymal cells could be either recruited from bone marrow (65) or normal breast stroma (66). In breast tumor cells, it has been identified that MSCs could be selectively recruited to the sites of growing carcinoma through cytokine such as IL-6 and CXCL7, where they interact with breast cancer CSCs (65, 66). In addition, MSCs are capable to differentiate into CAFs as well as adipocytes, which also interact with cancer cells (67).

CAFs have been demonstrated to have the ability to support tumorigenesis by stimulating angiogenesis, cell proliferation and invasion (68). CAFs in breast tumors expressed high levels of IL-6 (68, 69), which mediated epithelial-stromal interactions and promoted tumorigenesis (70). CAFs were reported to induce trastuzumab resistance in HER2 positive breast cancer cells (71). More importantly, IL-6 could in turn reactivate breast stromal fibroblasts through STAT3-dependent manner (72). CAFs could affect intra tumoral CD8+ and FoxP3+ T cells via IL-6 in tumor microenvironment (73). Recent findings also indicated miR-149’s role in the crosstalk between tumor cells and CAFs, which highlighted the potential therapeutic strategy using interfering miRNAs (74). There was growing evidence support that CAFs promote stem cell-like properties of hepatocellular carcinoma via IL-6/STAT3/Notch signaling pathway (75).

In a recent study, a novel developed liposomal nanoparticle loaded with anti-IL6R antibody which deliver to tumor microenvironment achieved a significant effect in inhibiting the metastasis of breast cancer cells in mouse models (76).

Obesity has been recently identified as a negative prognostic factor in breast cancer (77, 78), which appears to be independent of menopausal status, tumor stage, and hormone-related factors (79). According to the reported literature, adipocytes produced inflammatory cytokines such as IL-6 in obesity individuals (80). IL-6 was reported to mediate crosstalk between preadipocytes and breast ductal carcinoma *in situ* cells which may lead to progression of early-stage breast cancer (81). In addition, adipose-derived stem cells (ADSCs) promoted tumor initiation and accelerated tumor growth through IL-6 production (82). Obesity was suggested to induce resistance to anti-VEGF therapy in breast cancer by up-regulating IL-6 (83).

## IL-6’s Functional Role in Breast Cancer Development

### Experimental Studies

The predominant role of IL-6 in cancer is its key promotion of tumour growth. It has been demonstrated that deregulated IL-6 signaling pathway plays important roles in proliferation, migration, and adhesion among tumors (84–87). High level of IL-6 in breast cancer tissues stimulated Jagged-1 expression to promote cell growth and maintain the aggressive phenotype (88). High level of IL-6 secretion may facilitate tumor cell growth *via* suppressing apoptosis and promoting angiogenesis (89). High expression of IL-6Rα was also demonstrated to induce apoptosis resistance in breast cancer (90). In metastatic lesions of breast cancer patients, upregulated IL-6 was identified which may lead to chemotherapy resistance such as paclitaxel (91). The crosstalk between adipocytes and breast cancer cells in cancer progression has attracted much attention in recent years. The adipocyte-derived IL-6 was reported to promote breast cancer metastasis by inducing PLOD2 expression through activating the JAK/STAT3 and PI3K/AKT signaling pathways (92). In a recent study on triple-negative breast cancers (TNBCs), restraining of IL-6 and IL-8 expressions prominently suppressed both *in vitro* and *in vivo* cancer cell proliferation (93).

IL-12, which is produced by activated antigen presenting cells including dendritic cells and macrophages, was reported to inhibit tumor development (94). Some studies suggested that high expression level of IL-12 receptor were found to significantly increase breast cancer patients’ survival, especially in the more aggressive subtypes (95). It is also critical to initiate the differentiation of naive CD4+ T cells to T helper type 1 (Th-1) cells (96). However, the correlation between IL-6 and IL-12 remains elusive in breast cancer. According to the reported literature, the Th-1/Th-2 imbalance plays important role in the development of breast cancer (97). And circulating Th-1 and Th-2 levels and their ratios are associated with ER-negative and TNBC, suggesting their contribution in breast cancers (98). IL-6 played dual functions on Th-1/Th-2 differentiation by promoting Th-2 differentiation and inhibiting Th-1 polarization simultaneously (29).

IL-6 is a vital player during acute inflammation, controlling not only the inflammatory response but also tissue metabolism (99). Under chronic inflammation circumstance, IL-6 may induce cachexia through cytokines production and metabolism change in both lipids and proteins (100). Over-expression of IL-6 has been proved to be related with atrophy by promoting muscle protein metabolism (101). Cachexia and its related diseases account for approximately one third of all cancer-related deaths (102). Inflammatory breast cancer (IBC) describes a highly aggressive form of breast cancer of diverse molecular subtypes and clonal heterogeneity. The signature of IBC is recognized by its inflammation feature which is associated with IL-6 expression. A recent study published in May 2022 revealed that IL-6 signaling stimulate cell proliferation in IL-6R and HER2-expressing responsive sub-clones in IBC, and this effect was abrogated by the IL-6R neutralizing antibody Tocilizumab (103).

IL-6 is able to diffuse through cells structures and tissues in tumor microenvironment due to its low molecular weight (104). Tumor microenvironment-associated inflammation, mainly regulated by cytokines including IL-6, has been well-documented to contribute to every stage of cancer progression (105–108). Accumulating evidence has proved the significance of senescent cells in the microenvironment of cancer cells, of which pro-inflammatory IL-6 and IL-8 are consistently present. In this study, IL6 was reported to induce a self-reinforced senescence/inflammatory milieu responsible for the epithelial plasticity and stemness features which prone to a more aggressive phenotype in breast cancer (109).

Despite significant therapeutic achievements have been made in recent years, breast cancer is still one of the most common cancers with high mortality in women worldwide. Estrogen receptor (ER) α-positive breast cancers account for more than two thirds of all the category and endocrine therapies such as selective and aromatase inhibitors remain the standard adjuvant therapy for these tumors. However, majority of patients will develop drug resistance after treatment for several years and alternative hormone therapy is needed afterwards (110, 111). Interestingly, IL6/STAT3 signaling was suggested to drive metastasis in ER positive breast cancer independent of ER, decoupling IL6/STAT3 and ER oncogenic pathways could sensitize some hormonal resistant patients (112). In another study, similar conclusion was reported that Tocilizumab, an antibody that binds to IL-6R, could robustly reverse tamoxifen resistance (113). In compliance with this result, clinical breast cancer samples analysis confirmed that IL-6R expression was significantly associated with tamoxifen resistance in breast cancer tissues, with high IL-6R expression correlated with poor survival (113). Apart from the role in ER positive breast cancer, IL-6 was identified to trigger the migration and invasion of ER negative breast cancer cells *via* activation of YAP signals (114).

IL-6 could upregulate circulating VEGF in breast cancer patients, which was confirmed to promote angiogenesis and metastasis (115). Downregulation of IL-6 was related to the better response to breast cancer therapy (11, 116). Ligation of IL-6 with IL-6R activates Janus kinase (JAK) tyrosine kinases leading to phosphorylation of signal transducer and activator of transcription 3 (STAT3), which is a well-studied cancer signaling pathway. Moreover, the expression level of IL-6 was higher in aggressive tumors with multi-drug resistance and is negatively related to the expression of estrogen receptor in breast cancer patients (117, 118). Recently, the fact that IL-6-mediated Jagged1/Notch signaling pathway enhanced the ability for breast cancer cells metastasis has been demonstrated (119). All the evidence suggested that IL-6 and its receptor as attractive therapeutic targets.

### Clinical Studies

In many preclinical models, IL-6 has been demonstrated to promote carcinogenicity, angiogenesis and metastasis (88, 118, 120, 121). IL-6 has been implicated in resistance to trastuzumab treatment in HER2 positive patients. The induction of IL-6 inflammatory feedback loop leads to the expanded population of CSCs, which lead to high levels of this cytokine secretion. The addition of tocilizumab, an anti-IL-6R antibody, was reported to be capable for the interruption against this feedback loop (122). Based on this finding, a Phase I clinical trial started from 2017 with combined treatment including trastuzumab and tocilizumab for patients with metastatic trastuzumab-resistant HER2+ breast cancer was carried out (NCT03135171). According to the reported literature, IL-6 signaling is a major determinant of TNBC cell proliferation and viability (123), and this chemotherapy-associated inflammatory cytokine may promote resistance mechanisms in TNBC cells as well (124). A Phase Ib/II, open-label, multicenter, randomized umbrella study is being carried out to evaluate the efficacy and safety of multiple immunotherapy-based treatment combinations including tocilizumab in patients with metastatic or inoperable locally advanced TNBC (NCT03424005).

### The Prognostic Significance of IL-6 and Its Correlation With Survival

The prognostic impacts of preoperative IL-6 expression levels in patients with breast cancer remain controversial. In a meta-analysis extracted from thirteen articles containing 3,224 breast cancer patients showed that IL-6 expression was not associated with lymph node metastasis, tumor size, or histologic grade. Moreover, there was no correlation between IL-6 expression and disease-free survival. However, the combined hazard ratio for OS was 2.15 (125). Another study included 1,380 patients with early-stage invasive breast cancer revealed that high IL-6 expression is associated with better disease-free survival and breast cancer specific survival (126). However, anther investigation involving 55 female patients with invasive breast cancer demonstrated that the individuals with IL-6 ≥10.0 pg/ml had poorer overall survival compared with those with IL-6 <10.0 pg/ml (127). Similarly, it was reported that high level of serum IL-6 secreted by metastatic breast cancer cells were correlated with poor survival (15). Regarding the roles of IL-6 in ER positive breast cancers as previously described, we further summarized the prognostic value of IL-6 among different subtypes of breast cancer patients (**Table 1**). For example, in a prospective study included 240 patients who underwent surgery for management of newly diagnosed breast cancer, the associations between plasma concentration of IL-6 and breast cancer recurrence during a six-year follow-up period were examined. The result showed that patients with recurrence had higher levels of circulating IL-6 only among those with HER2 negative tumors. Results of survival analyses revealed an association of high levels of IL-6 with poor recurrence-free survival in patients with HER2 negative and TNBC patients (132).

**Table 1:** Prognositc value of IL-6 in different types of breast cancers.

Tumor subtype^a^	Prognostic value of IL-6	Reference
Luminal A	•ER+ breast cancer cells express and/or secrete lower cytokine levels than ER- cells (128, 129) •High levels of gene expression of IL-6 receptor in luminal A and B (130)	(128–130)
Luminal B	•The luminal B HER2+ group was found to feature the highest spontaneous secretion of IL-6 among subgroups (131) •High levels of gene expression of IL-6 receptor in luminal A and B (130)	(130, 131)
HER2 (+/-)	•HER2- patients with recurrence had higher levels of circulating IL-6 (P=0.024) (132) •High IL-6 expression was significantly associated with DFS in HER2- (P = 0.026) (126) •High serum in HER2+ patients (P<0.05) (133) •IL6 as good indicator in both HER2- (P = 0.001) and HER2+ subgroups (P = 0.002) (134) •Association with HER2 or endocrine therapy resistance (122, 135)	(122, 126, 132–135)
TNBC	•Patients with recurrence had higher levels^b^ of circulating IL-6 (P=0.024) (132) •High IL-6 expression was significantly associated with DFS in non-TNBC (P = 0.003) (126) •Induction of TNBC progression (123, 136, 137)	(123, 126, 132, 136, 137)
ER/PR status	•High IL-6 expression was significantly associated with DFS in ER+ (P = 0.025) (126) •High serum in ER+ patients (P<0.05) (133) •IL6 as the independent prognostic factor for good outcome (P=0.001) (134)	(126, 133, 134)
Metastasis	•Higher serum IL-6 level correlated with more metastatic sites (P<0.0001) (15)	(15)

a Luminal A (ER+ and/or PR+, HER2-, and Ki-67 index<15%); luminal B ([ER+ and/or PR+, HER-, and Ki-67 index≥15%] or [ER+ and/or PR+,and HER2+]); HER2 only (ER-, PR-, and HER2+); TNBC (ER-, PR-, and HER2-).

b High and low levels were determined based on the median value.

The approximate percentage of HER2 gene amplified in human breast cancer is 25%, which is characterized by a more aggressive phenotype (138). Trastuzumab, as one of the targeted therapeutic agents for HER2+ breast cancer patients, has totally changed the treatment course. Although many patients benefit from the HER2 targeted therapy, nearly half of them will develop drug resistance after one to two years of treatment (139). Evidence showed that overexpression of HER2 in breast CSCs increased IL-6 production, which could promote CSC self-renewal. The fact that HER2 targeted therapy could prominently activate the IL-6 inflammatory loop and expand the CSC population, signified the cause of IL-6 in Herceptin resistance (122). In ER-negative breast cancer, findings demonstrated that IL-66/Stat3/NF-κB inflammatory loop was activated (140). And it has been proved that leptin-induced STAT3 is partially cross activated through SK1-mediated IL-6 secretion and gp130 activation, suggesting the potential significance of this pathway (141).

A growing body of evidence indicated Bazedoxifene, which is a synthetic anti-gp130 compound, could effectively disrupt the IL-6R/gp130 interactions thus inhibit cell viability, and overall cell survive, proliferation as well as cell migration in TNBC (142). A novel in-house prepared IL-6 pathway inhibitor namely 6a, which is capable of selectively inhibiting STAT3 activation following IL-6 stimulation in MDA-MB-231 breast cancer (143). Sarilumab, an FDA-approved anti-IL-6R antibody for rheumatoid arthritis, which blocks both mIL-6R and sIL-6R, is currently under clinical studies for breast cancer (144). Siltuximab, which is a neutralizing anti-IL-6 antibody, delayed engraftment of MCF-7 humanized xenograft tumors and elicited tumor xenograft regression in tumors (145). The anti-IL-6 receptor antibody, Tocilizumab, is effective in the treatment of various autoimmune diseases such as rheumatoid arthritis (RA) (146). Experimental results demonstrates that IL-6 pathway targeted drugs may have additional benefit in HER2+ breast cancer (122). It has been proved that IL-6 receptor inhibitor suppressed bone metastases in a breast cancer cell line (147). Another study showed that IL-6R antagonist Tocilizumab significantly decreases breast cancer stem cell and inhibits tumor growth in Notch3-expressing breast cancers (148). The high level of IL-6R expression in spindle-shaped stromal cells such as CAF was not associated with the vasculature but could be used as prognostic determinant of early breast cancer (149). CAFs in tumor microenvironment played a vital role in developing trastuzumab resistance by magnifying CSCs bulge and activating multiple pathways (150). Regarding this, combination of anti-IL-6 antibody, or multiple pathway inhibitors with trastuzumab maybe novel strategy to reverse drug resistance in HER2+ breast cancer (71). Genotype of IL-6 was prominently related to early events among patients bearing with ER-negative tumors (151). The IL-6 signaling loop mediated drug resistance to PI3K inhibitors *via* inducing epithelial-mesenchymal transition (EMT) and CSCs expansion in human breast cancer cells (152). In summary, IL-6 signaling pathway may be potential treatment target for breast cancer patients in the future. The previously mentioned agents targeting the IL-6/IL-6R signaling for breast cancer therapy were listed in **Table 2**.

**Table 2:** Agents directly targeting the IL-6/IL-6R/gp130 complex for breast cancer therapy.

Agents	Antibody/Compound	Preclinical	Clinical trial	Mechanism
Bazedoxifene	Synthetic Anti-gp130 compound	Inhibit the growth of IL-6-induced SUM159 breast cancer cell line (153)	Breast tissue density change (NCT00774267) (NCT00418236)	1. Inhibition STAT3 phosphorylation by disrupting IL-6/gp130 interface (153) 2. Estrogen antagonist in breast tissue
6a	Anti-IL-6 synthetic pyrrolidinesulphonylaryl compound	Inhibition of STAT3 phosphorylation in IL-6 stimulated MDA-MB-231 breast cancer cell line (143)		Selective inhibition of STAT3 phosphorylation (143)
Sarilumab	IL-6R antagonist		To eliminate minimal residual disease in TNBC (NCT04333706)	Selective inhibition of STAT3 phosphorylation (143)
Siltuximab	CNTO-328, IL-6 mAb which received FDA-approval	Treatment in 6 orthotopically implanted PDX lines *in vivo* (145)		To prevent binding to soluble and membrane bound interleukin-6 receptors
Tocilizumab	IL-6R antagonist	Trastuzumab-resistant breast tumor xenograft mouse model	For metastatic HER2 positive breast cancer resistant to Trastuzumab (NCT03135171) Treatment combinations in patients with metastatic or inoperable locally advanced TNBC (NCT 03424005)	

IL-6 could promote the response of acute phase inflammatory *via* increasing the production of acute inflammatory proteins. IL-6 was also correlated with elevated CRP in different kinds of cancers including breast cancer (154), renal cancer (155), lung cancer (156), and colorectal cancer (157). Although breast cancers rarely are characterized by inflammation, a growing body of evidence nevertheless suggests that inflammatory process also play an important role in breast cancer progression (158, 159). Based on the reported literature, the results from epidemiologic studies in different centres are conflicting, with some showing significant association between elevated CRP levels and poor prognosis in breast cancers while others show no association (160–162). In a study consisted of 700 women with early-stage breast cancer found that elevated levels of CRP measured 2.5 years after diagnosis were associated with reduced DFS and OS (163). Similarly, another investigation included 2,910 women for up to seven years after invasive breast cancer diagnosis revealed elevated CRP levels were significantly associated with reduced DFS and OS (164). Preoperative CRP level was indicated as a more accurate prognostic factor compared with other factors, such as histological grade, tumor factor and node factor (127).

## Conclusions

**Figure 1:**
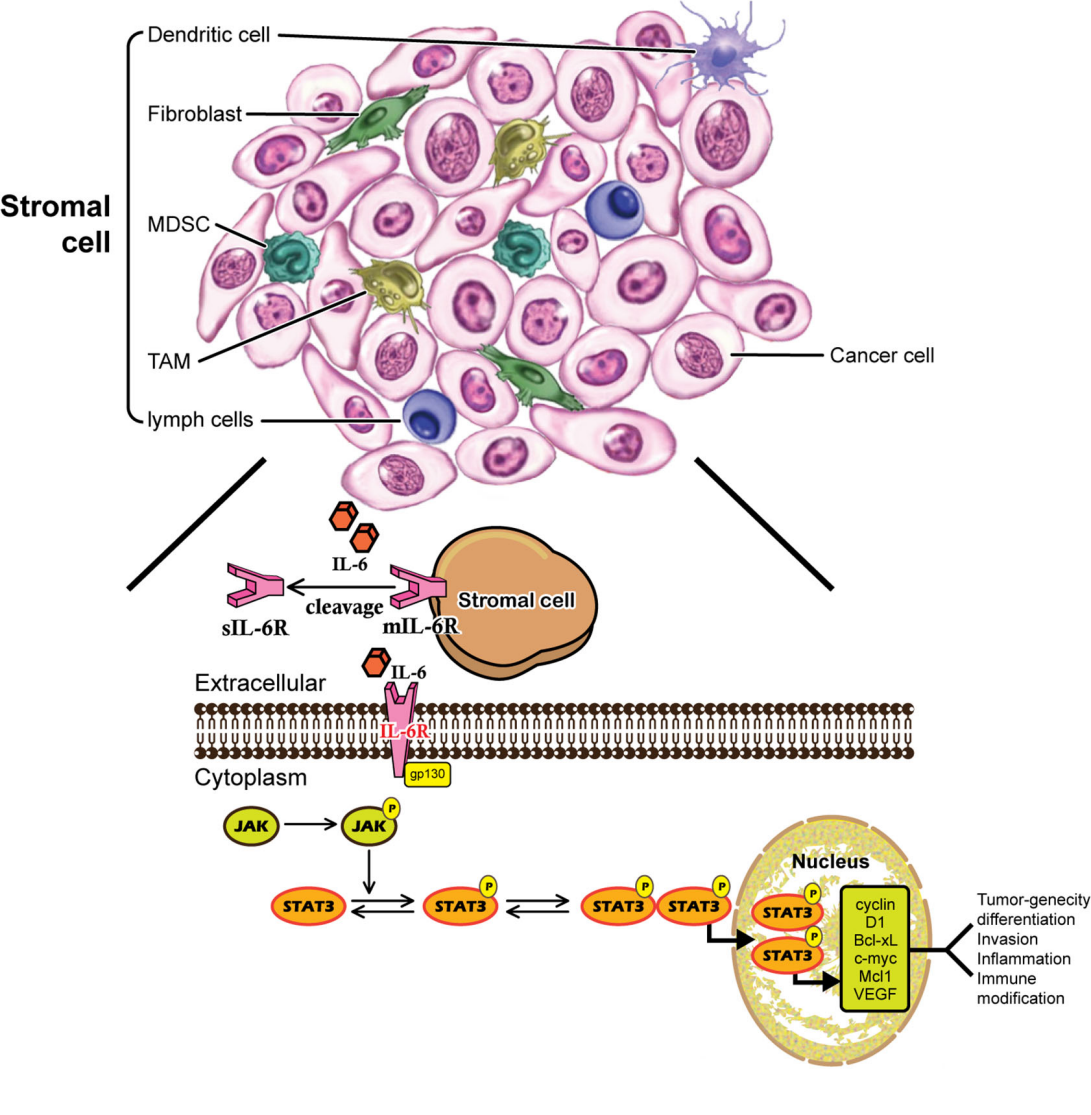
The interaction network of IL-6 and breast cancer cells/stromal cells.

IL-6 is a pleiotropic cytokine in the regulations of various physiological and pathological processes. IL-6 causes uncontrolled inflammatory responses resulting in chronic inflammation and even carcinoma. IL-6 expression is associated with poor prognosis for breast cancer. The interaction network of IL-6 in breast cancer cells/stromal cells is listed as **Figure 1**. The IL-6 signal transduction pathway including IL-6, IL-6R, sIL-6R, gp130, JAK, and STAT3 has been suggested as promising therapeutic targets for breast cancer. Several antibodies for IL-6/IL-6R have been developed, either as single drug or combined with other traditional chemotherapy, have demonstrated dramatical outcome in both preclinical and clinical trials. In addition to the critical roles of IL-6/JAK/STAT3 signaling in breast cancer, hyperactivation of this pathway has also been implicated in suppressing anti-tumor immune responses in tumor microenvironment. Treatments targeting the IL-6/JAK/STAT3 pathway have provided benefit for patients with breast cancer by directly inhibiting tumor cell growth and activating anti-tumor immunity. Taken together, strategy targeting the IL-6/JAK/STAT3 signaling pathway, which has already been shown to be beneficial in certain cancers including breast cancer, has proven to be effective. Combination of IL-6 signaling pathway inhibitor and other targets blockage drugs may serve as novel strategy to treat IL-6 mediated immune disease and human cancers.

## Author Contributions

Conception or design of the work, JC and YW. Data collection, JWC, WY, QH, YC, and KZ. Drafting the article, JC and JWC. Critical revision of the article, YW. All authors contributed to the article and approved the submitted version.

## Conflict of Interest

The authors declare that the research was conducted in the absence of any commercial or financial relationships that could be construed as a potential conflict of interest.

## Publisher’s Note

All claims expressed in this article are solely those of the authors and do not necessarily represent those of their affiliated organizations, or those of the publisher, the editors and the reviewers. Any product that may be evaluated in this article, or claim that may be made by its manufacturer, is not guaranteed or endorsed by the publisher.
